# For infants with surgical necrotizing enterocolitis, does primary anastomosis or stoma formation provide shorter parenteral nutrition?

**DOI:** 10.1038/s41372-025-02555-z

**Published:** 2026-01-12

**Authors:** Cherise Brackett, Pavel Chernyavskiy, Brynne Sullivan

**Affiliations:** 1 Division of Neonatology, Department of Pediatrics, University of Virginia School of Medicine, Charlottesville, VA USA; 2 Division of Biostatistics, Department of Public Health Sciences, University of Virginia School of Medicine, Charlottesville, VA USA

**Keywords:** Intestinal diseases, Outcomes research

## Methods

### Design

The STAT Trial was a multicenter, multinational randomized controlled trial conducted at 12 centers across Europe, Canada, South America, Singapore, and New Zealand from April 2010 to January 2019 [1]. The study was approved by the Institute of Child Health/Great Ormond Street Hospital Research Ethics Committee/National Research Ethics Service (09/H0713/58), and each institution obtained individual ethical approval. The trial was registered with the International Standard Randomized Controlled Trial Register (ISRCTN): ISRCTN01700960. Pediatric surgeons obtained written consent before surgery. However, enrollment and randomization occurred during laparotomy after each infant was assessed for findings of necrotizing enterocolitis (NEC) and the extent of NEC. Infants who were determined to benefit from either intervention were then included in the study.

### Allocation

Randomization occurred intraoperatively after inspecting the bowel for exclusion criteria below. A weighted minimization method was then used to evaluate how assigning an infant to each intervention could affect the balance of the following factors:

1. Weight at enrollment (<1000 g, 1000–2000 g, >2000 g)
2. Mechanical ventilation
3. Inotropic support
4. Focal vs. multifocal disease
5. Area of intestinal involvement

This approach maintains randomness while reducing confounding by assigning each new participant to keep the trial arms as balanced as possible.

### Blinding

Blinding was not possible as randomization occurred during laparotomy when disease findings and extent of disease were assessed.

### Follow-up period

Patients were evaluated in the surgery clinic at 3 months and 1 year.

### Participants

Seventy-nine infants were enrolled following parental consent. Each site received individual ethics approval.

### Inclusion criteria

Suspected necrotizing enterocolitis (NEC) requiring laparotomy due to perforation or failed medical management.

### Exclusion criteria

Exclusions included:

- no NEC
- focal intestinal perforation
- extensive NEC requiring resection, likely to result in short bowel syndrome
- NEC isolated in the colon with high bleeding risk
- intraoperative instability
- Consent declined

### Intervention

Following resection of necrotic bowel, infants underwent either primary anastomosis or formation of a stoma.

### Outcomes

The primary outcome was defined as the duration of parenteral nutrition in days. Secondary outcomes included mortality, need for additional surgery, and intestinal complications (e.g., anastomotic leak, stoma-related complications, obstruction, recurrent NEC).

### Analysis and sample size

A sample size of 66 provided 80% power to detect a 14-day difference in time to full enteral feeds, with 13 more added for anticipated 20% mortality. An interim analysis was performed after recruiting 40 participants, halfway to the goal enrollment.

## Results

Gestational age, birth weight, and sex were similar between groups, with no difference in bowel involvement or extent of NEC.

### Primary outcome

Median duration of parenteral nutrition was significantly shorter in the primary anastomosis group (30 days; range 4–105) compared to the stoma group (51 days; range 3–310) (*p* < 0.05).

### Secondary outcomes

Mortality rates were similar between groups. Intestinal complications were more frequent in the stoma group (46%) versus the anastomosis group (16%) (*p* < 0.05).

#### Study conclusion

At laparotomy for NEC, when there is no disease distal to the resected intestine, primary anastomosis enhances the recovery from NEC, reduces the risk of multiple intestinal complications and does not increase adverse outcomes.

##### Commentary

NEC remains one of the most serious gastrointestinal emergencies in the neonatal intensive care unit, primarily affecting premature infants. Risk factors include prematurity, low birth weight, antibiotic exposure, and formula feeding, while protective factors include breastfeeding, feeding protocols, and probiotics [2–5]. Infants requiring surgery for NEC face the highest risks of poor outcomes, including death, short bowel syndrome, and long-term nutritional dependence [6].

The optimal surgical approach, whether to perform primary anastomosis or stoma formation, has remained a debated issue [7]. This trial is the first randomized controlled study to provide evidence on this topic, addressing a significant gap in the literature.

The multinational nature of the STAT Trial, covering 12 centers, supports generalizability. However, 73% of participants came from just three countries (Canada, the UK, and Serbia). There may be potential differences in concurrent practices between locations that could modify the effect of the surgical approach. The absence of a center in the primary analysis may have weakened the ability to account for practice variation or demographics in determining the effect of surgical approach.

Similarly, the lack of detail on feeding strategies post-operatively limits the interpretation of the primary outcome (duration of parenteral nutrition). Feeding protocols across institutions often differ in duration of small volume feeds before advancing, speed of advancing to full volume feeds, and timing of re-introduction of feeds after surgical NEC [8, 9]. These protocols directly impact the duration of parenteral nutrition, the study’s primary outcome. In addition, while the sample size was calculated to detect a difference in days to full feeds, the analysis used days on parenteral nutrition as the primary outcome. This is a subtle distinction. Infants may achieve full feeds but still require parenteral nutrition in the setting of poor weight gain from presumed malabsorption. In this case, the time to full feeds is shorter than the timeframe of requiring parenteral nutrition.

Furthermore, while randomization was designed to balance illness severity, surgeon discretion in selecting cases for randomization may have introduced selection bias, limiting the generalizability. Demographic characteristics of the infants who were not included in the study are not provided. It is unclear how this group differs from those included in the study. The study was not powered for subgroup analysis, such as examining outcomes in extremely low birth weight or critically ill infants who are at higher risk of mortality after surgical NEC [10].

Despite these limitations, the STAT Trial provides compelling evidence that primary anastomosis may lead to quicker recovery in terms of parenteral nutrition needs, without an increase in complications or mortality.

## Discussion

The STAT Trial is a randomized controlled trial addressing the surgical management of NEC in neonates. It provides the first high-quality evidence supporting primary anastomosis over stoma formation in select infants, showing a shorter median duration of parenteral nutrition and fewer complications without an increase in mortality.

While limitations such as regional enrollment concentration, lack of feeding protocol data, and potential selection bias exist, the trial’s findings represent an advancement. Future research should explore whether these findings hold true in extremely preterm or critically ill infants and evaluate long-term outcomes such as growth, neurodevelopment, and quality of life.

### EBM lesson: Cox regression and proportional hazards

This study utilized Cox regression to assess the risk of achieving full enteral nutrition. Descriptive statistics showed a difference in median duration of parenteral nutrition, and Cox regression estimated a hazard ratio (HR) accounting for the time to event and potential confounders.

#### Why Cox Regression?

Cox regression is a common method for time-to-event (survival) analysis in clinical research [11]. It allows adjustment for confounders and handles censored data (e.g., patients who die before completing the event). In this study, the outcome, completion of parenteral nutrition, was right-censored for deaths or last follow-up.

The independent variable was the intervention (anastomosis vs. stoma), while covariates included those used in the minimization criteria (weight, ventilation, inotropes, disease distribution, and bowel segment involved), which attempt to balance the treatment groups. The final model included the intervention group and preoperative inotrope requirement, further protecting against any confounding if imbalances in inotrope use remained after minimization.

#### Hazard ratios

Hazard ratios quantify the relative instantaneous event rates of achieving the event, in this case, stopping parenteral nutrition. A hazard ratio >1 indicates a higher rate of achieving the event in the intervention group (primary anastomosis). For example, a hazard ratio of 1.1 would indicate that, at any point in time, the treated group would have a 10% increased event rate compared to a control. In contrast, a relative risk of 1.1 reflects a 10% increased odds of the event over the entire study.

#### Advantages of hazard ratios

Compared to odds ratios or relative risks, hazard ratios retain more information about the timing of events and can account for censoring [12]. Censoring refers to the inability to observe the exact time to an outcome due to confounders such as death or study duration. In the STAT Trial, censoring refers to infants whose time to achieving enteral independence was not observed due to death, or they remained on parenteral nutrition at the time of study conclusion.

#### Limitations of hazard ratios

Despite its strengths, Cox regression can be influenced by selection bias, particularly in the context of mortality. If one intervention group has a higher mortality before the outcome can be achieved, the remaining survivors may be inherently different. This bias can distort comparisons. Kaplan-Meier curves, as used in the STAT Trial, help visualize time-to-event differences and how hazard ratios evolve.

## Data Availability

There are no limits on data availability and materials. For access to data and materials from the original STAT Trial, please contact the relevant authors.
